# Supplementary material for: Adapting a Plant Tissue Model to Animal Development: Introducing Cell Sliding into VirtualLeaf
Source: Bull Math Biol. 2019 Mar 29;81(8):3322–41. doi: 10.1007/s11538-019-00599-9 (PMC6677868; doi:10.1007/s11538-019-00599-9)
Supplement: Supplementary file 1 — Supplementary material 1 (pdf 141 KB) [file 11538_2019_599_MOESM1_ESM.pdf]

5 Supplementary Material

Supplementary Text S1

Detailed description of the algorithms, the parameter files, and the initial conditions.

Video S1

Differential-adhesion driven cell rearrangement in VirtualLeaf. Cell mixing as in Figure 2A. Initial condition: 200 green and 200 red cells of $A_T = A(0) = 100$. $J(\text{green}, \text{green}) = J(\text{red}, \text{red}) = 20$, $J(\text{red}, \text{green}) = 10$, $J(\text{cell}, \text{medium}) = 30$; $l_{\text{min}} = 6$, $l_{\text{max}} = 8$, $\Delta x = 0.5$; $T = 10$. Simulation length: 500,0000 Monte Carlo Steps (MCS)

Video S2

Differential-adhesion driven cell rearrangement in VirtualLeaf. Engulfment as in Figure 2B. Initial condition: 200 green and 200 red cells of $A_T = A(0) = 100$. $J(\text{green}, \text{green}) = 20$, $J(\text{red}, \text{red}) = 10$, $J(\text{red}, \text{green}) = 20$, $J(\text{green}, \text{medium}) = 20$, $J(\text{red}, \text{medium}) = 40$. Simulation length: 500,0000 Monte Carlo Steps (MCS)

Video S3

Differential-adhesion driven cell rearrangement in VirtualLeaf. Cell sorting as in Figure 2C. Initial condition: 200 green and 200 red cells of $A_T = A(0) = 100$. $J(\text{green}, \text{green}) = 20$, $J(\text{red}, \text{red}) = 10$, $J(\text{red}, \text{green}) = 30$, $J(\text{cell}, \text{medium}) = 30$. Simulation length: 500,0000 Monte Carlo Steps (MCS)

Video S4

Differential-adhesion driven cell rearrangement in VirtualLeaf. Incomplete cell sorting with only T1 transitions as in Figure 2D. $\theta_{\text{T1}} = 0.25$; other parameters as in Video S3. Simulation length: 500,0000 Monte Carlo Steps (MCS)

Video S5

Effect of interface specific cortical tension. Simulation with cell-type-specific cortical tension applied only at cell medium interfaces as in Figure 3, top-left panel. $P_T(\text{red}) = 20$ at cell-medium interfaces and $P_T(\text{green}) = 20$

at cell-medium interfaces. All other parameters have default values (see Supporting Text S1). This figure shows the tissues after a simulation of 500,000 MCS.

Video S6

Effect of interface specific cortical tension. Simulation with cell-type-specific cortical tension applied only at cell medium interfaces as in Figure 3, bottom-right panel. $P_T(\text{red}) = 40$ at cell-medium interfaces and $P_T(\text{green}) = 40$ at cell-medium interfaces. All other parameters have default values (see Supporting Text S1). This figure shows the tissues after a simulation of 500,000 MCS.

Video S7

Effect of interface specific cortical tension. Simulation with cell-type-specific cortical tension applied only at cell medium interfaces as in Figure 3, bottom-right panel. $P_T(\text{red}) = 40$ at cell-medium interfaces and $P_T(\text{green}) = 20$ at cell-medium interfaces. All other parameters have default values (see Supporting Text S1). This figure shows the tissues after a simulation of 500,000 MCS.

Video S8

Simulation of epithelial cell packing Case I with T1 transitions and straight walls; $\lambda_{\text{cortical}} = 10$, $J(\mathbf{e} \to L, \mathbf{e} \to R) = 500$. MCS 0 to 40000 with stride 500; cell colors indicate number of neighbors as in Figure 4C-D.

Video S9

Simulation of epithelial cell packing Case II with T1 transitions and straight walls; $\lambda_{\text{cortical}} = 26$, $J(\mathbf{e} \to L, \mathbf{e} \to R) = 0$. MCS 0 to 40000 with stride 500; cell colors indicate number of neighbors as in Figure 4C-D.

Video S10

Simulation of epithelial cell packing Case III with T1 transitions and straight walls; $\lambda_{\text{cortical}} = 26$, $J(\mathbf{e} \to L, \mathbf{e} \to R) = -3560$. MCS 0 to 40000 with stride 500; cell colors indicate number of neighbors as in Figure 4C-D.

Video S11

Simulation of epithelial cell packing Case I with sliding and flexible walls; $\lambda_{\text{cortical}} = 10$, $J(\mathbf{e} \to L, \mathbf{e} \to R) = 500$. MCS 0 to 40000 with stride 500; cell colors indicate number of neighbors as in Figure 4C-D.

Video S12

Simulation of epithelial cell packing Case II with sliding and flexible walls; $\lambda_{\text{cortical}} = 26$, $J(\mathbf{e} \to L, \mathbf{e} \to R) = 0$. MCS 0 to 40000 with stride 500; cell colors indicate number of neighbors as in Figure 4C-D.

Video S13

Simulation of epithelial cell packing Case III with sliding and flexible walls; $\lambda_{\text{cortical}} = 26$, $J(\mathbf{e} \to L, \mathbf{e} \to R) = -3560$. MCS 0 to 40000 with stride 500; cell colors indicate number of neighbors as in Figure 4C-D.

Video S14

Effect of cell resolution on cell sorting kinetics. Left, control simulation of cell mixing (cf. Figure 2A) with default values of $l_{\min} = 6$ and $l_{\max} = 8$; Right, refined simulation of cell mixing with reduced values of $l_{\min} = 3$ and $l_{\max} = 4$ such that twice the number of edges and nodes is used for each cell. Bottom panel shows the summed length of red-green cell-cell interfaces relative to the total length of all cell-cell interfaces in the configuration,

$$\frac{1}{\sum_{\mathbf{e} \in E} \|\mathbf{e}\|} \sum_{\{\mathbf{e} \in E | \mathbf{e} \text{ is red-green interface}\}} \|\mathbf{e}\|, \tag{5}$$

as a function of time. The moving dot indicates the present time.
